# Supplementary material for: Comprehensive Expression Analysis of the WRKY Gene Family in Phoebe bournei under Drought and Waterlogging Stresses
Source: Int J Mol Sci. 2024 Jul 2;25(13):7280. doi: 10.3390/ijms25137280 (PMC11242546; doi:10.3390/ijms25137280)
Supplement: Supplementary file 1 [file ijms-25-07280-s001.zip › ijms-3065120-supplementary.pdf]

Supplementary Materials

Figure S1: Different development stages under drought stress in *P. bournei*;

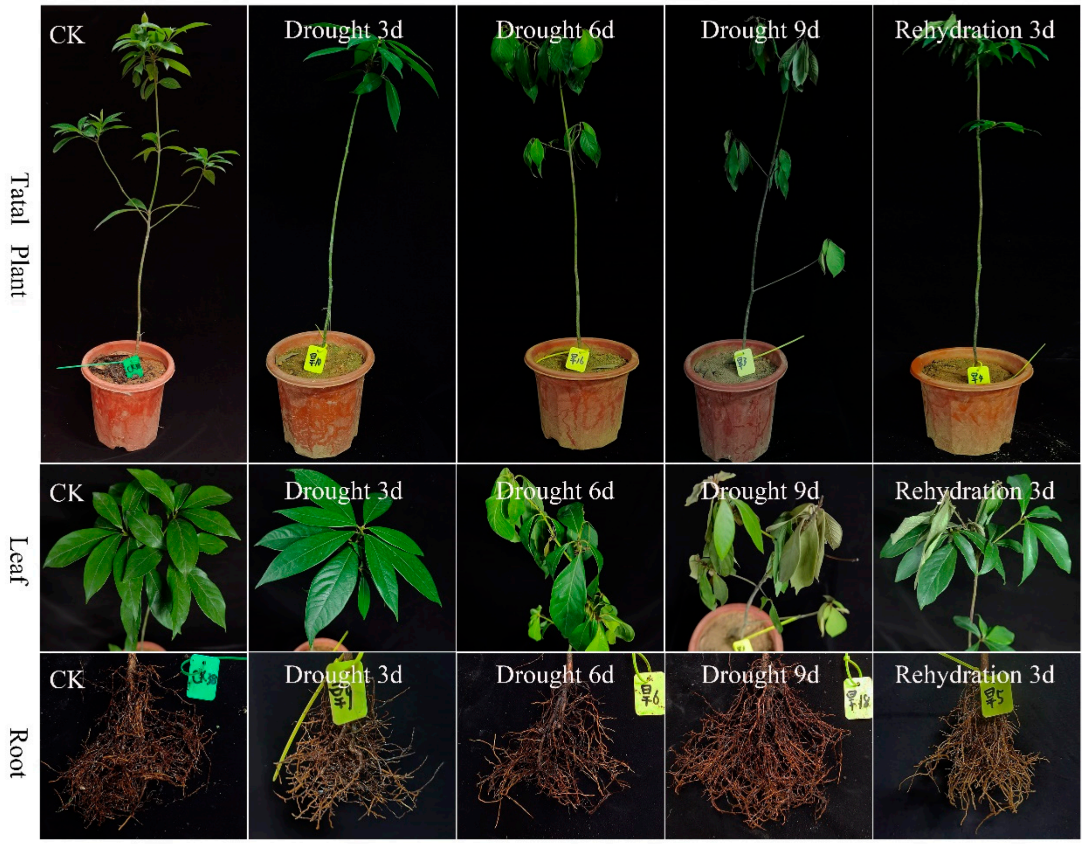

Figure S2: Different development stages under waterlogging stress in *P. bournei*;

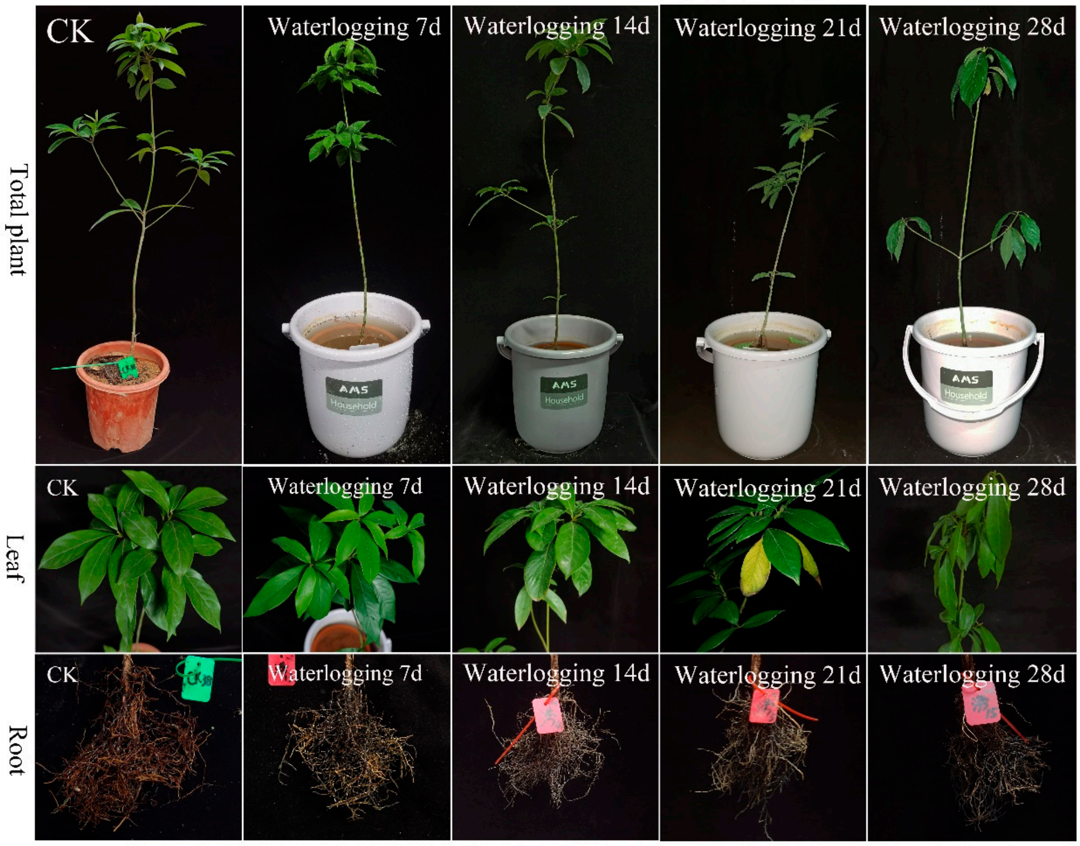

Table S1: The cDNA first strand synthesis reaction system;

Reagent	Usage amount (μL)
RNA template	2.0
8 * gDNA remover	2.0
RNase-free ddH2O	12.0
5 * RT SuperMix	4.0
Total	20.0

The temperature and time of the reverse transcription process are carried out in the following order: the first maintain 37 °C, lasting for 15 minutes; the second maintain 85 °C lasts for 5 seconds; the third annealing to 4 °C. Finally, the cDNA samples were stored

in a refrigerator at -20 °C.

Table S2: The RT-qPCR system.

Reagent	Usage amount (μL)
2*S6 Universal SYBR qPCR mix	10.0
Primer-F (10μm)	0.8
Primer-R (10μm)	0.8
cDNA template	2.0
ddH2O	6.4
Total	20.0

The specific reaction of the qPCR program is as follows: first, pre denature at 95 °C for 30 s, then denature at 95 °C for 5 s, and finally anneal at 60 °C for 34 s. The reaction ends after 40 cycles.
